# Factors associated with the use of cannabis for self-medication by adults: data from the French TEMPO cohort study

**DOI:** 10.1186/s42238-024-00230-2

**Published:** 2024-04-10

**Authors:** Solène Wallez, Isabelle Kousignian, Irwin Hecker, Selma Faten Rezag Bara, Astrid Juhl Andersen, Maria Melchior, Jean-Sébastien Cadwallader, Murielle Mary-Krause

**Affiliations:** 1 Sorbonne Université, INSERM, Institut Pierre Louis d’Epidémiologie et de Santé Publique, IPLESP, Equipe de Recherche en Epidémiologie Sociale, ERES, Paris, 75012 France; 2 Université Paris Cité, Unité de Recherche « Biostatistique, Traitement Et Modélisation Des Données Biologiques » BioSTM - UR 7537, 75006 Paris, France; 3 Sorbonne Université, Faculté de Médecine Saint-Antoine, Département de Médecine Générale, Paris, 75012 France; 4 Sorbonne Université - Faculté de Médecine, Site Saint-Antoine, UMR-S 1136 – N° BC 2908, Équipe Cohorte TEMPO, 27 Rue Chaligny, 75012 Paris, France

**Keywords:** Cannabis use for self-medication, Marijuana, Adults, Cohort, Cannabis use trajectories, Associated factors

## Abstract

**Background:**

Medical cannabis, legalized in many countries, remains illegal in France. Despite an experiment in the medical use of cannabis that began in March 2021 in France, little is known about the factors associated with the use of cannabis for self-medication among adults.

**Methods:**

Data came from the French TEMPO cohort and were collected between December 2020 and May 2021. Overall, 345 participants aged 27–47 were included. Cannabis for self-medication was defined using the following questions: ‘Why do you use cannabis?’ and ‘In what form do you use cannabis?’. The penalized regression method “Elastic net” was used to determine factors associated with the use of cannabis for self-medication, with the hypothesis that it is mainly used for pain in individuals who have already used cannabis.

**Results:**

More than half of the participants reported having ever used cannabis (58%). Only 10% used it for self-declared medical reasons (*n* = 36). All self-medication cannabis users, except one, were also using cannabis for recreational purposes. The main factors associated with cannabis use for self-medication vs. other reasons included cannabis use trajectories, the presence of musculoskeletal disorders, tobacco smoking, and parental divorce.

**Conclusions:**

Engaging in cannabis use during adolescence or early adulthood may increase the likelihood of resorting to self-medication in adulthood. Due to the propensity of individuals with cannabis use during adolescence to resort to uncontrolled products for self-medication, this population should be more systematically targeted and screened for symptoms and comorbidities that may be associated with cannabis use.

**Supplementary Information:**

The online version contains supplementary material available at 10.1186/s42238-024-00230-2.

## Background

In the European Union, cannabis is the most widely used illicit drug, with a prevalence of 27.3% among adults aged 15–64 (European Monitoring Centre for Drugs and Drug Addiction 2021). Legislation regarding cannabis use has evolved in many countries, and its use, especially for medical purposes, is now authorized. Many states in the United States, as well as Canada, Israel and some European countries, allow the prescription of cannabis for specific indications (Abuhasira et al. 2018). In recent years, cannabis use among middle-aged and older adults (aged ≥ 50 years or ≥ 65 years) has significantly increased (Salas-Wright et al. 2017; Han et al. 2017; Solomon et al. 2021) and has increased more rapidly compared to all other age groups (Bobitt et al. 2019). One study showed that 52.1% of individuals aged 50 and over reported using cannabis for medical purposes, compared to only 37.3% of individuals aged 30 to 49 and 17.8% for 18 to 29-year-old (Sexton et al. 2019). Additionally, medical cannabis use increased between 2013 and 2015 in both states that legalized medical cannabis use and those that did not (Han et al. 2018). Nevertheless, the most important factor that shifted older adults’ beliefs about cannabis and contributed to their decision to begin using cannabis at an advanced age, especially for medical purposes, is the legalization (Baumbusch and Sloan 2022).

Among medical cannabis users, pain is the most cited reason (Rotermann and Pagé 2018; Park and Wu 2017), and anxiety, depression, post-traumatic stress disorder (PTSD), and insomnia are also frequently cited (Azcarate et al. 2020; Kvamme et al. 2021). Cannabis is used as a substitute for other prescription pain medications, including opioids (Sexton et al. 2016). Nonetheless, cannabis use disorder was more often reported among adults with pain compared to those without (Hasin et al. 2020).

In France, cannabis is illegal, even for medical purposes, and it has one of the most repressive illegal drug legislation among European countries (European Monitoring Centre for Drugs and Drug Addiction 2021). Nevertheless, cannabis use prevalence is high: 47.3% of 18–64-year-olds have experimented with cannabis, 10.6% used it over a 12-month period, 5.9% over a 30-day period, 3.0% are regular users, and 1.7% are daily users (Nézet et al. 2022). In March 2021, France launched an experimentation regarding the medical use of cannabis, which will last until 2023. The clinical trial includes five therapeutic indications: neuropathic pain refractory to available treatments (e.g., post-traumatic neuropathic pain, post-surgical pain, chronic sciatica polyneuropathy, post zoster pain, phantom pain, spinal cord pain, multiple sclerosis pain, post-stroke pain), forms of severe epilepsy resistant to drugs, certain rebellious symptoms in oncology or symptoms in palliative situations (e.g., pain, fatigue, nausea-vomiting, sleep disorders, anxiety, loss of appetite, sadness), and painful spasticity accompanying central nervous system diseases, such as multiple sclerosis (Agence Nationale de Sécurité du Médicaments et des produits de santé (ANSM) 2021).

With such high prevalence and recent evolutions, understanding factors associated with medical use of cannabis becomes a major public health issue. Medical cannabis use was found to be associated with non-medical use (Park and Wu 2017), higher frequencies of use (Rotermann and Pagé 2018; Woodruff and Shillington 2016; Lin et al. 2016), non-cannabis drug use (Lin et al. 2016), poorer health (Rotermann and Pagé 2018; Lin et al. 2016), the use of psychoactive pharmaceuticals, including sedatives or tranquilizers (e.g., Xanax), stimulants (e.g., Ritalin), and opioid analgesics (e.g., fentanyl) (Rotermann and Pagé 2018), psychological problems (Woodruff and Shillington 2016), less willingness to change consumption (Woodruff and Shillington 2016) and living in lower-income households (Rotermann and Pagé 2018). Yet, medical cannabis users also exhibit a lower frequency of drug problems and a tendency to be low-risk users compared to moderate-severe users (Woodruff and Shillington 2016), and lower levels of alcohol use disorders (Lin et al. 2016).

To our knowledge, no study has examined the associated factors with cannabis use for self-medication in countries where it remains illegal, nor have they taken into account trajectories of use from adolescence to adulthood. In this context, the aim of this study is to identify factors associated with the use of cannabis for self-medication among adults in France, specifically those related to childhood, family, or individual events influencing individuals' cannabis self-medication. We hypothesize that it is mainly used for pain in individuals who are already accustomed to using cannabis, for whatever reason.

## Methods

### Design and setting of the study

**Fig. 1:**
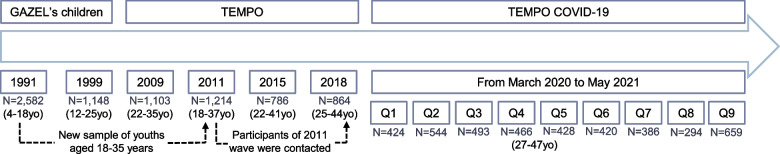
Timeline of TEMPO data collection and number of participants from 1991 to 2021

The TEMPO (Trajectoires EpidéMiologiques en POpulation) study was set up in 2009 among French adults aged 22 to 35 years who were previously followed in 1991 and 1999 as part of a study on children’s psychological problems (Mary-Krause et al. 2021), and whose parents had participated in the GAZEL cohort (Goldberg et al. 2015). In 2009, participants were interviewed about their mental health and psychoactive substance use, including cannabis use. Subsequent interviews took place in 2011, 2015, 2018, and nine times in 2020 and 2021 in the context of the COVID-19 pandemic (Fig. 1).

**Fig. 2:**
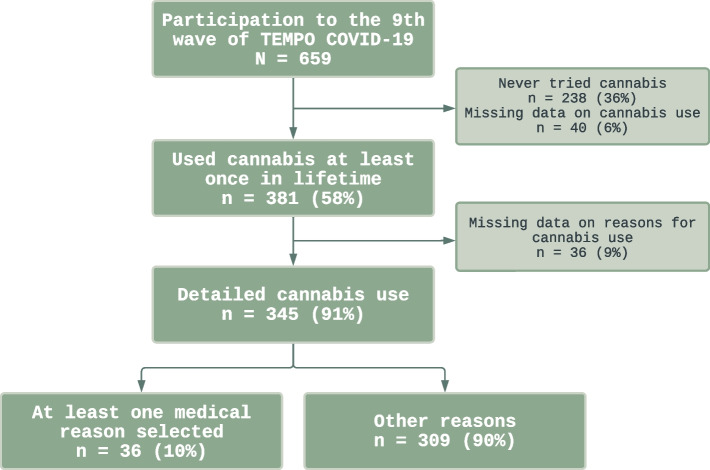
Flowchart of the population included in the study from TEMPO cohort participants

Data for this study were collected in the final wave of TEMPO COVID-19, spanning from December 2020 to May 2021, during which detailed information regarding cannabis use and associated reasons were reported for the first time. The questionnaire was completed by 659 TEMPO cohort participants, among whom 381 reported having ever used cannabis, and 345 answered questions about reasons and forms of cannabis use (Fig. 2).

### Measures

All measures of cannabis use were self-reported. History of cannabis use and childhood characteristics data were collected from TEMPO cohort questionnaires completed between 2009 and 2018. GAZEL cohort data were used to describe parental characteristics.

#### Outcome: use of cannabis for self-medication

The use of cannabis for self-medication was defined based on the questions “Why do you use cannabis?” and “In what form do you use cannabis?”. These questions were posed during the last wave of the study, which took place from December 2020 to May 2021. Based on the scientific literature (Haug et al. 2017; Reinarman et al. 2011; Ogborne et al. 2000), 12 reasons and four forms were selected to define use of cannabis for mental or physical health reasons (Additional file 1). Thus, individuals who selected at least one of these items were considered to be using cannabis for self-medication purposes, even if they also had so-called recreational use (“cannabis use for self-medication” vs. “cannabis use for other reasons”).

The medical reason “to get asleep” is missing in the list of proposed reasons, even though it is a frequent reason (Azcarate et al. 2020; Kvamme et al. 2021). However, respondents had the option to add an "other" response at the end of the list and specify the modality. Out of the 29 individuals who used this option, only four mentioned sleep, one of them being already included in the medical cannabis category. To avoid biasing the results with potentially heterogeneous responses, we did not include these subjects in the main analysis. Nevertheless, we conducted a sensitivity analysis including this reason.

Another sensitivity analysis was performed with a second definition of self-medication with cannabis, including additional items that could be related to mental or physical health, particularly focusing on the depressive state or sadness (Additional file 1), following the results of the qualitative study (Rezag Bara et al. 2023).

#### Potentially associated factors

Factors associated with medical cannabis use, as described in the literature, such as sociodemographic characteristics (Rotermann and Pagé 2018), cannabis use (Rotermann and Pagé 2018; Woodruff and Shillington 2016; Lin et al. 2016), mental or physical health (Rotermann and Pagé 2018; Lin et al. 2016), and the use of psychoactive substances (Lin et al. 2016), as well as other potentially associated factors with cannabis use such as negative events during childhood (Rezag Bara et al. 2023) and parental characteristics (Boden et al. 2020), were studied in the analyses.

##### Sociodemographic characteristics

Sex (“female”; “male”), age (continuous), marital status (“married, in civil union, in relationship”; “single, divorced, widowed”), and having children (“no”; “yes”) were included in the analysis. The socioeconomic position of individuals was determined by combining educational level (“ > bachelor's degree”; “ ≤ bachelor's degree”), employment category (“executive, intermediate profession, craftsman, merchant, company manager”; “employee, worker”), type of contract (“permanent or civil servant”; “fixed-term contract, internship, volunteer, apprentice, subsidized contract, or undeclared work”), and main activity prior to lockdown (“employed or student”; “unemployed”). Each of these variables was coded as 0 or 1, summed, and dichotomized by the lowest quartile (“intermediate or high”; “lower”) (Aljandaleh et al. 2020; Redonnet et al. 2012).

##### Cannabis use

**Fig. 3:**
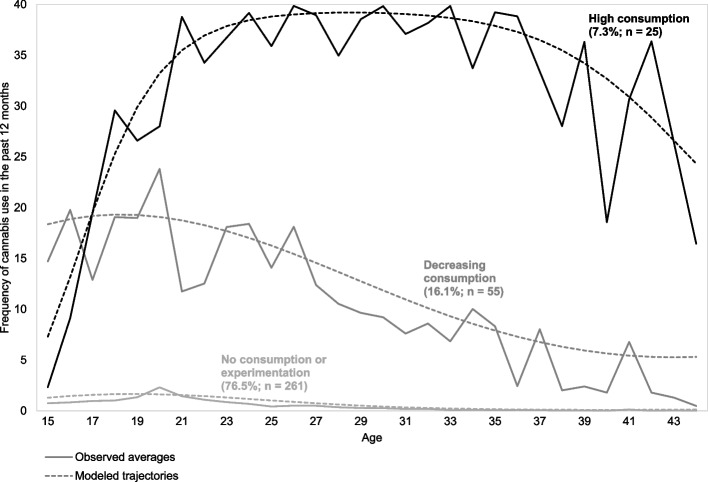
Estimation of the mean cannabis use trajectories (TEMPO cohort study, 1999–2018, France, *n* = 341)

Lifetime, past year, and past month cannabis use frequencies were asked to determine current or former use (Spilka et al. 2018). The frequency of cannabis use during the year was also asked in 1999, 2009, 2011, 2015, and 2018. This information allowed us, using the Group-Based Trajectory Modeling (GBTM) method (Nagin and Odgers 2010), to determine longitudinal trajectories of cannabis use from adolescence to adulthood among participants who completed at least one study questionnaire. Following recommendations to choose the number of trajectories and polynomial orders (Jones et al. 2001), three trajectories were determined, and each participant was assigned to a trajectory (“experimentation”; “decreasing consumption”; “high consumption”) (Fig. 3). The Cannabis Abuse Screening Test (CAST) was used to identify a high risk of problematic cannabis use (“no”; “yes”) (Legleye et al. 2007). Participants were asked about the age of cannabis initiation in each wave since 1999, the earliest information available was used. The variable was then recoded into 2 categories (“late initiation”; “early initiation”) considering a cut-off at 16 or lower (Hayatbakhsh et al. 2013; Melchior et al. 2017). Participants were asked if they used cannabis alone (“no”; “yes”).

##### Mental health

Participants' mental health disorders, including anxiety, depression and internalizing symptoms, were determined using the standardized DSM-oriented Adult Self-Report (ASR) scale (Achenbach 2015). Following DSM-V criteria, all responses were summed, and the score was then standardized with a mean of 50 and a standard deviation of 10 (T score), and then dichotomized by the 85th percentile (“no”; “yes”).

##### Physical health

Medical conditions were self-reported using the following question: “Have you ever been diagnosed by a doctor with any of the following health problems?”. Individuals could choose from a list of 22 pathologies (“no”; “yes”): accidental polytrauma, arthritis, asthma, cancer, cardiovascular disease (including heart murmur, …), chronic digestive disease (ulcer, …), cranial trauma, Crohn’s disease or other inflammatory bowel disease, diabetes, endometriosis, epilepsy, high blood pressure, insomnia, migraine, multiple sclerosis, musculoskeletal disorders (lumbago, sciatica, tendinitis, …), nervous breakdown, overweight or obesity, post-traumatic stress disorder, psychiatric disorders, and rheumatoid arthritis.

##### Use of psychoactive substances

Two statements have been used for smoking status (“non-smoker”; “regular, occasional or ex-smoker”). Initiation of tobacco consumption was considered early if it occurred before the age of 14 years old (Clergue-Duval et al. 2019; Breslau and Peterson 1996). Participants’ alcohol consumption in the past 12 months was defined using four categories: “Never, once a month or less”, “2 to 4 times a month”, “2 to 3 times a week”, and “4 times a week or more”. The use of 7 other illicit drugs (i.e. ecstasy, hallucinogens, stimulants, cocaine, non-prescription drugs, heroin, or solvents) was asked and grouped into a variable corresponding to lifetime experimentation with at least one of them (“no, never”; “yes, at least once”).

##### Negative events during childhood

Events before the age of 15 included repeating a grade (“no”; “yes”) and violence during childhood (“no”; “yes”). This variable was defined using three retrospective questions about events experienced during childhood, addressed in the 2011 TEMPO wave: "Have you witnessed serious arguments or violent behavior between your parents?", "Has anyone tried to denigrate, devalue, or make fun of you in an insistent, hurtful way (e.g., at school)?", and "Have you suffered from a serious lack of affection?".

##### Parental characteristics

Parental smoking was defined by the highest level of smoking of either parent: non-smoker, former-smoker, and smoker. This information was obtained directly from one parent in the GAZEL study (1989–2015) and from the other parent in the TEMPO study (2009 or 2011), and the highest risk category was retained. The level of parental alcohol use (“non-unhealthy alcohol use”; “unhealthy alcohol use”), parental divorce or separation before the participant's 17th birthday (“no”; “yes”), and parental depression (“no”; “yes”) were reported by parents in the GAZEL study (1989–2015).

### Statistical analyses

Participants’ characteristics were described according to cannabis use: “cannabis use for self-medication” vs. “cannabis use for other reasons”. Differences between the two groups were tested using Pearson's Chi-squared test, Fisher's exact test, or Wilcoxon rank sum test.

To determine factors associated with the use of cannabis for self-medication, we used logistic regression modelling. All factors found to be associated with cannabis use for self-medication in the literature were tested in univariate logistic models. Due to the high number of variables with a *p*-value < 0.2 relative to the total number of subjects (Peduzzi et al. 1996), the penalized regression method “Elastic net” was used to perform a selection of the adjustment variables and to address the issue of multi-collinearity (Zou and Hastie 2005; Tibshirani 1996). We then conducted a sensitivity analysis, including other negative life events.

The number of participants with missing data for each variable of interest varied from four (1.2%), for the cannabis use trajectory, to 14 (4.1%), for smoking status and insomnia. In order to include all participants who reported cannabis use in multivariate model, all covariates were imputed using the Multiple Imputation by Chained Equations (MICE) by Fully Conditional Specification (FCS) (White et al. 2011; Bodner 2008), using 20 datasets (Graham et al. 2007).

Multivariate logistic regression was then used to identify associated factors, and one sensitivity analyse was carried out by expanding the definition of cannabis use for self-medication. Interactions between selected variables were tested.

Data processing and analyses were performed using SAS® 9.4 and R 4.1.0 software (R Core Team 2020) (packages *dplyr* (Hadley et al. 2021), *glmnet* (Jerome et al. 2010), *mice* (van Buuren and Groothuis-Oudshoorn 2011), *gtsummary* (Sjoberg et al. 2021)). 

## Results

### Participants description

Among the 345 participants aged 27–47 included in our study, 36 (10%) said they had used cannabis for self-medication reasons. Nevertheless, all except one also checked off recreational reasons. Among these self-medication users, the most cited reason for using cannabis was to manage stress (58.3%), followed by managing anxiety (36.1%) and managing headaches, migraines, or treating chronic pain (11.1%) (Additional file 1). Comparisons of participants’ characteristics according to reasons for cannabis use are presented in Table 1. The majority of the participants (61.4%) were females, the median age was 41.1 years, and 71.8% had an intermediate or high socioeconomic position.

**Table 1:** Characteristics of TEMPO cohort participants included in the study by reason for cannabis use

Variable*, n (%)*	Total (*n* = 345^1^)	Cannabis use for other reasons *n* = 309)	Cannabis use for self-medication (*n* = 36)	*p*-value^2^
*Sociodemographic characteristics*				
**Sex**				0.960
Female	212 (61.4%)	190 (61.5%)	22 (61.1%)	
Male	133 (38.6%)	119 (38.5%)	14 (38.9%)	
**Age***, median (IQR)*	41.1 (38.4, 43.5)	41.1 (38.5, 43.5)	40.7 (37.8, 42.6)	0.335
**Marital status**				0.550
Married, in civil union, in relationship	289 (84.0%)	260 (84.4%)	29 (80.6%)	
Single, divorced, widowed	55 (16.0%)	48 (15.6%)	7 (19.4%)	
**Having children**	263 (76.7%)	240 (78.2%)	23 (63.9%)	0.055
**Socioeconomic position**				**0.005**
Intermediate or high	242 (71.8%)	224 (74.2%)	18 (51.4%)	
Lower	95 (28.2%)	78 (25.8%)	17 (48.6%)	
*Cannabis use*				
**Current or former cannabis use**				** < 0.001**
Experimenters (at least once in lifetime)	267 (80.4%)	254 (85.5%)	13 (37.1%)	
Used in the past year	28 (8.4%)	22 (7.4%)	6 (17.1%)	
Use in the past month	37 (11.1%)	21 (7.1%)	16 (45.7%)	
**Cannabis use trajectory**				** < 0.001**
Experimentation	261 (76.5%)	246 (80.7%)	15 (41.7%)	
Decreasing consumption	55 (16.1%)	43 (14.1%)	12 (33.3%)	
High consumption	25 (7.3%)	16 (5.2%)	9 (25.0%)	
**High risk of problematic cannabis use**^**3**^	22 (7.5%)	11 (4.2%)	11 (33.3%)	** < 0.001**
**Age of onset cannabis**				0.081
Late initiation (after 16 years)	240 (70.0%)	220 (71.4%)	20 (57.1%)	
Early initiation (16 years old and under)	103 (30.0%)	88 (28.6%)	15 (42.9%)	
**Using cannabis alone**	50 (14.7%)	29 (9.5%)	21 (58.3%)	** < 0.001**
*Mental health*				
**Anxiety**	40 (11.6%)	32 (10.4%)	8 (22.2%)	0.051
**Depression**	46 (13.4%)	36 (11.7%)	10 (27.8%)	**0.016**
**Internalized disorders**	43 (12.6%)	35 (11.5%)	8 (22.2%)	0.105
*Physical health*				
**Overweight or obesity**	49 (14.6%)	40 (13.3%)	9 (25.7%)	**0.049**
**Chronic digestive disease**	12 (3.6%)	10 (3.3%)	2 (6.1%)	0.337
**Cancer**	7 (2.1%)	6 (2.0%)	1 (3.0%)	0.522
**Asthma**	30 (9.0%)	27 (9.0%)	3 (9.1%)	1.000
**Migraines**	56 (16.8%)	49 (16.3%)	7 (21.2%)	0.471
**Nervous breakdown**	48 (14.5%)	40 (13.4%)	8 (25.0%)	0.107
**Musculoskeletal disorders**	104 (31.3%)	86 (29.0%)	18 (51.4%)	**0.007**
**Arthritis**	35 (10.4%)	32 (10.6%)	3 (9.1%)	1.000
**High blood pressure**	16 (4.7%)	14 (4.6%)	2 (5.9%)	0.669
**Insomnia**	48 (14.5%)	38 (12.8%)	10 (29.4%)	**0.017**
**Crohn’s disease**	12 (3.6%)	9 (3.0%)	3 (9.1%)	0.103
**Multiple sclerosis**	2 (0.6%)	1 (0.3%)	1 (3.0%)	0.188
**Post-traumatic stress disorder**	8 (2.4%)	6 (2.0%)	2 (6.1%)	0.179
**Cranial trauma**	16 (4.8%)	14 (4.7%)	2 (6.1%)	0.666
*Use of psychoactive substances*				
**Smoking status**				**0.001**
Non-smoker	135 (40.8%)	130 (44.1%)	5 (13.9%)	
Regular, occasional or ex-smoker	196 (59.2%)	165 (55.9%)	31 (86.1%)	
**Age of smoking initiation**				**0.022**
Late initiation (after 14 years old)	192 (62.1%)	177 (64.4%)	15 (44.1%)	
Early initiation (14 years old and under)	117 (37.9%)	98 (35.6%)	19 (55.9%)	
**Alcohol consumption**				0.205
Never, once a month or less	80 (23.3%)	69 (22.5%)	11 (30.6%)	
2 to 4 times a month	105 (30.6%)	96 (31.3%)	9 (25.0%)	
2 to 3 times a week	93 (27.1%)	87 (28.3%)	6 (16.7%)	
4 times a week or more	65 (19.0%)	55 (17.9%)	10 (27.8%)	
**Other drugs lifetime use**^**4**^				**0.045**
No, never	268 (78.8%)	245 (80.3%)	23 (65.7%)	
Yes, at least once	72 (21.2%)	60 (19.7%)	12 (34.3%)	
*Negative events during childhood*				
**Repeating a grade**	162 (49.2%)	142 (48.3%)	20 (57.1%)	0.323
**Violence during childhood**	128 (41.2%)	110 (39.6%)	18 (54.5%)	0.098
*Parental characteristics*				
**Tobacco parents**				0.194
Non-smoker	93 (27.2%)	87 (28.4%)	6 (16.7%)	
Former-smoker	129 (37.7%)	111 (36.3%)	18 (50.0%)	
Smoker	120 (35.1%)	108 (35.3%)	12 (33.3%)	
**Alcohol parents**				**0.002**
Non-unhealthy alcohol use	237 (69.3%)	220 (71.9%)	17 (47.2%)	
Unhealthy alcohol use	105 (30.7%)	86 (28.1%)	19 (52.8%)	
**Parental divorce before 17 years old**	18 (5.3%)	12 (3.9%)	6 (16.7%)	**0.007**
**Parental depression before 17 years old**	39 (11.4%)	31 (10.1%)	8 (22.2%)	**0.047**

^1^Due to missing values, categories do not always add up to 345

^2^Obtained by Pearson's Chi-squared test, Fisher's exact test or Wilcoxon rank sum test (for continuous variable)

^3^According to the Cannabis Abuse Screening test (CAST)

^4^Including ecstasy, hallucinogens, stimulants, cocaine, non-prescription drugs, heroin, or solvents

Overall, 19.6% of participants used cannabis in the past 12 months, with 56.9% of them using it in the past 30 days (Table 1), and 36.9% using it regularly (Rotermann and Pagé 2018). Additionally, three cannabis use trajectories were identified (Fig. 3): experimentation (76.5%), decreasing consumption (16.1%) and high consumption (7.3%).

### Modelling cannabis use for self-medication

The main factors retained by the penalized regression method “Elastic net” for the logistic regression with cannabis for self-medication as the outcome were socioeconomic position, cannabis use trajectories, musculoskeletal disorders, insomnia, smoking status and parental divorce before age 17. Having used cannabis or currently using it increased the odds of self-medicating with cannabis by at least four, just as having musculoskeletal disorders increased the odds by 2.5, compared to other reasons to use. Even though not statistically significant, smoking tobacco and experiencing parental divorce during childhood were associated with higher odds of self-medication with cannabis (Table 2). No interactions were found between factors included in analyses.

**Table 2:** Factors associated with cannabis use for self-medication by logistic regression (TEMPO cohort study, 2020–2021, France, *n* = 345)

Variable^3^	Univariate model		Multivariate imputed^1^ model		1st Sensitivity analysis^2^		2nd Sensitivity analysis^3^	
	OR [95% CI]^4^	*p*-value	OR [95% CI]^4^	*p*-value	OR [95% CI]^4^	*p*-value	OR [95% CI]^4^	*p*-value
**Socioeconomic position**								
Intermediate or high	reference		reference		reference		reference	
Lower	2.71 [1.32, 5.54]	**0.006**	1.77 [0.80, 3.94]	0.159	1.75 [0.81, 3.78]	0.155	1.26 [0.74, 2.14]	0.402
**Cannabis use trajectory**								
Experimentation	reference		reference		reference		reference	
Decreasing consumption	4.58 [1.98, 10.4]	** < 0.001**	4.36 [1.77, 10.7]	**0.001**	3.79 [1.58, 9.10]	**0.003**	5.28 [2.72, 10.2]	** < 0.001**
High consumption	9.22 [3.43, 24.3]	** < 0.001**	6.07 [1.98, 18.6]	**0.002**	6.69 [2.27, 19.8]	**0.001**	5.45 [2.06, 14.5]	**0.001**
**Musculoskeletal disorders**								
No	reference		reference		reference		reference	
Yes	2.60 [1.28, 5.32]	**0.008**	2.49 [1.09, 5.67]	**0.030**	2.28 [1.03, 5.05]	**0.041**	1.87 [1.10, 3.19]	**0.021**
**Insomnia**								
No	reference		reference		reference		reference	
Yes	2.84 [1.21, 6.27]	**0.012**	2.11 [0.80, 5.51]	0.129	2.23 [0.89, 5.63]	0.088	1.49 [0.74, 2.96]	0.260
**Smoking status**								
Non-smoker	reference		reference		reference		reference	
Regular, occasional or ex-smoker	4.88 [2.01, 14.6]	**0.001**	2.72 [0.95, 7.81]	0.063	2.40 [0.90, 6.40]	0.080	1.13 [0.67, 1.88]	0.649
**Parental divorce before 17 years old**								
No	reference		reference		reference		reference	
Yes	4.88 [1.60, 13.6]	**0.003**	3.28 [0.94, 11.5]	0.063	2.84 [0.82, 9.88]	0.100	1.29 [0.44, 3.80]	0.642

^1^Model imputed with MICE

^2^Reasons reported in the “other” category and related to “to get asleep” have been added to the definition of the use of cannabis for self-medication

^3^Reasons added to the definition of the use of cannabis for self-medication after qualitative interviews about depression/sadness: to forget about life’s problems, to be happy, to fill a void, to feel well

^3^Variables selected by Elastic net regression

^4^*OR* Odds Ratio, *95% CI* Confidence Interval

### Sensitivity analysis

In the first sensitivity analysis, the medical reason “to get asleep” was included in the definition. A total of 39 individuals were considered to use cannabis for self-medication. Results in the same way than those without this criterion were found (Table 2).

In the second sensitivity analysis, four additional reasons were included (Additional file 1). A total of 136 individuals (39.4%) were considered to use cannabis for self-medication, and the most cited reasons were to feel well (83.1%), to forget about life’s problems (20.6%), and to manage stress (15.4%). The results obtained using the new definition were consistent with those obtained previously (Table 2).

In the final sensitivity analysis, when incorporating additional information on negative life events into the multivariate regression (i.e., repeating a grade, violence during childhood, and parental depression before 17 years), parental divorce before 17 years old remains the highest odds (Additional file 2). Furthermore, odds ratios for cannabis use trajectories remained unchanged after including negative life events during childhood. When excluding cannabis use trajectories, odds-ratios associated with the included negative life events are not significant, except for parental divorce before 17 years old, which was nearly significant in the principal analysis. Additionally, without considering cannabis use trajectories, the smoking status became significant with a higher odds-ratio due to the almost systematic use of tobacco with cannabis in France (Le Nezet et al. 2021).

## Discussion

### Interpretation

This study highlighted that individuals with high or declining lifetime cannabis use trajectories and presenting musculoskeletal disorders were more likely to use cannabis for self-medication, in accordance with our hypothesis.

Understanding the reasons for using cannabis, in a country where its use remains illegal is essential. Our study confirms that exclusive use for self-medication is an exception, with mixed use (combining self-medication and recreational) remaining the most common, which is consistent with other studies (Rotermann and Pagé 2018; Furler et al. 2004). A review found that non-medical cannabis use was a common factor associated with medical use (Park and Wu 2017). Individuals using cannabis for medical reasons in a country where it is not allowed must have used or currently use it recreationally (Roy-Byrne et al. 2015), probably because it is easier to through known distribution channels and because knowing the therapeutic effects and benefits of cannabis use makes it a more viable option. Additionally, in a survey, 29% of participants discovered the benefits of medical cannabis as a result of recreational use (Swift et al. 2005). Conversely, risk perception is higher among individuals who have never used it (Kilmer et al. 2007).

Similar to other studies (Rotermann and Pagé 2018; Woodruff and Shillington 2016; Lin et al. 2016), we also found that individuals who used cannabis as teenagers or young adults are more likely to self-medicate with cannabis. This could be attributed to a normalization of cannabis use in this population and a lower risk perception (St-Jean et al. 2022), or it might be linked to cannabis dependence (Winters and Lee 2008). Moreover, medical cannabis use is more socially accepted than recreational use (Baumbusch and Sloan 2022), especially when prescribed by a health professional (Spilka et al. 2019). This increased awareness of the benefits and greater acceptance of cannabis use for self-medication may lead individuals who use cannabis to define their use as medical.

Some of the reasons listed to define the medical use of cannabis may be withdrawal symptoms. For instance, appetite change, weight loss, and physical discomfort may be a consequence of cannabis withdrawal syndrome (Budney et al. 2004) or a pre-existing reason for using cannabis (Furler et al. 2004). In this context, individuals with constant high lifetime cannabis use trajectories are more prone to experiencing pharmacological dependence and to report less cannabis use for medical reasons compared to those in declining lifetime cannabis use trajectories. As our results indicated the highest odds of using cannabis for self-medication among individuals with high cannabis consumption, the reasons listed to define medical cannabis use are not solely withdrawal symptoms.

Having musculoskeletal conditions, which generally result in persistent pain (Musculoskeletal conditions 2021) and affect morbidity, quality of life, and mortality (Beaudart et al. 2018) is a factor associated with medical cannabis use to reduce pain, with minor adverse effects (Furrer et al. 2021). Among medical cannabis users, pain is the most cited reason (Park and Wu 2017; Kvamme et al. 2021), with cannabis often used as a substitute for other pain treatments (Sexton et al. 2016), especially opioids (Kvamme et al. 2021; Boehnke et al. 2019), when conventional drugs have insufficient or undesirable effects (Kvamme et al. 2021; Krediet et al. 2020 Jul) despite an evident lack for efficacy (Riera et al. 2022). In a survey conducted in 15 European countries and Israel on chronic pain, 40% of participants reported inadequate management of their pain (Breivik et al. 2006). Using medical cannabis was found associated with poorer health (Rotermann and Pagé 2018; Lin et al. 2016) or is often used for multiple medical problems (Roy-Byrne et al. 2015), even some for which cannabis is not prescribed or licensed by the state (Bonn-Miller et al. 2014). Health literacy was found associated with substance use including cannabis (Kinnunen et al. 2022 Apr 1). Therefore, it is plausible that individuals who avoid conventional healthcare, possibly due to factors such as low levels of literacy, or experiences of stigma and discrimination, are more likely to engage in self-medication with cannabis. Unfortunately, we lack indicators of care avoidance.

In our study, discrepancies exist between self-declared medical conditions and the self-declared reasons for cannabis use. Among individuals using cannabis for self-medication, eight reported suffering from a nervous breakdown, but only three attributed their cannabis use to this reason. Of the four individuals who reported using cannabis to manage headaches, only half reported this symptom. However, considering that the question asks whether a doctor has formally diagnosed the condition, it becomes clear that more individuals are using cannabis for reasons not explicitly linked to medical problems. Using a diagnostic scale for anxiety, eight individuals were identified as anxious. Of these, five used cannabis to manage their anxiety, and three did not. Conversely, eight individuals reported using cannabis to relieve anxiety but did not receive a formal diagnosis according to the ASR.

Smoking status and parental divorce were nearly significantly associated with cannabis use for self-medication. In France, cannabis is mostly consumed in combination with tobacco. Indeed, 95% of those who smoked cannabis mixed it with tobacco (Le Nezet et al. 2021). Parental divorce before the age of 17 appears to be a proxy for a difficult or traumatic situation experienced during childhood, as adults with high cannabis use are more likely to have experienced negative or even highly traumatic childhood events (Boden et al. 2020).

### Limitations and strengths

Our study has several limitations to highlight. The TEMPO cohort is not representative of the general French population, given the way in which participants were recruited and the attrition. Thus, females and individuals with a higher socioeconomic position are overrepresented (Mary-Krause et al. 2021) and studies have shown that they use cannabis less (Le Nezet et al. 2021). However, the prevalence levels of cannabis experimentation are higher than national estimates in 2020 (57.8% vs. 46.1%) (Le Nezet et al. 2021). Self-reporting of cannabis use for self-medication may lead to underestimation or misclassification, as individuals might not be fully aware of their medical use and that they are using cannabis, for instance, to improve mood or alleviate certain symptoms. We did not specifically ask patients about which symptoms they independently address through cannabis use, but we employed the ASR anxiety symptom diagnostic scale, which allows to determine which symptoms they are experiencing. Among the eight individuals categorized with anxiety symptoms, five claim to use cannabis to manage anxiety, and this number rises to seven when including those who uses it to manage stress. This is also the reason why, two different definitions of self-medication with cannabis were tested by expanding the definition to include more ambiguous reasons between self-medication and recreational use, and the same results were found. Additionally, the limited number of individuals considered to have used cannabis for self-medication restricts the statistical power of the analysis and the number of variables included in the multivariate model. However, the use of Elastic net allows discarding the less contributing variables and ensures the consistency of the selection. Because of the sample size, individuals included in the analysis may be current users of cannabis or have used at some point in their lives, and the different reasons for use are only declared in the last questionnaire, without any precision on the chronology. It would have been interesting to study the evolution of the reasons to use cannabis with age. Moreover, the answers about reasons for use may have been retrospective, which may cause a memory bias. However, whenever possible, data were adjusted using previous information, such as experimentation, age of first use, or frequency of cannabis use. Additionally, within the proposed list of reasons for cannabis use, the category “to get asleep” is absent, despite its frequent citation (Azcarate et al. 2020; Kvamme et al. 2021). After incorporating the four individuals who mentioned sleep in the “other” possibilities, the results remained similar. Unfortunately, conditions such as epilepsy, for which the French National Agency for the Safety of Medicines and Health Products (ANSM) can deliver medicines containing cannabidiol (e.g., Epidyolex) (Reda et al. 2021), could not be included in the analyses as no individuals with this pathology were included in the sample. Indeed, Epidyolex prescription is limited to Lennox-Gastaut syndrome, which is a very rare and refractory condition in children, making their occurrence unidentifiable in a limited general population sample. Finally, because of the sample size and the legislation regarding cannabis, only one person reported using it solely for self-medication purposes, so we mostly compare recreational users only and mixed users.

These weaknesses are countered by the strengths of the study. Individuals were recruited regardless of their substance use, resulting in a heterogeneous population concerning cannabis use. Longitudinal follow-up provides prospective information for over 30 years, particularly on events experienced during childhood, mental and physical health, and on the level of use of the various substances. A considerable amount of information on the socio-demographic characteristics, health, and substance use of the participants' parents is also available. Asking participants about their reasons for using cannabis, rather than whether they use medical or recreational cannabis, allows for further reflection on the definition of self-medication with cannabis and limits an important bias, as medical cannabis may have multiple definitions depending on the individual. Moreover, taking into account the medical reasons from the literature in the initial analysis and then trying to integrate other borderline reasons into the definition helps confirm the self-medication classification. Using trajectories of cannabis use over a 19-year period captured individuals' use from adolescence to adulthood, rather than a single point in time. Finally, the use of elastic net and cross-validation makes it possible to combine the lasso and ridge penalties to regularize and select the variables and fit the logistic regression.

## Conclusion

Given the high prevalence of cannabis use, understanding the reasons for its use is crucial. Engaging in cannabis use during the formative periods of adolescence or early adulthood may increase the likelihood of resorting to self-medication in adulthood. This association suggests that in a country where cannabis use remains illegal, the most common way to learn about its health benefits and to obtain it is to first use it recreationally before switching to self-medication. Medical advice and support could contribute to a more informed and effective use of medical cannabis. However, a paper from 2020 suggested that individuals were less likely to disclose their use to their doctor when living in states where medical cannabis was illegal (Azcarate et al. 2020). Due to the propensity of individuals who used cannabis during adolescence to resort to uncontrolled products for self-medication, this population should be more systematically targeted and screened for symptoms and co-morbidities that may be associated with cannabis use. Further research into controlled cannabis products for symptom relief, as well as the formulation of policies advocating the regulation of these products, could potentially mitigate the use of illicit cannabis products whose health benefits remain questionable. This study underscores the need for a comprehensive understanding of the long-term implications and motivations associated with cannabis consumption across different life stages. Additional research is needed to better understand the reasons for self-medication with cannabis, its forms of use, but above all, its efficacy and safety.

### Supplementary Information

**Supplementary Material 1.**

**Supplementary Material 2.**

## Data Availability

Due to the personal questions asked in this study, research participants were guaranteed that the raw data will be remain confidential. On reasonable request including standards for General Data Protection Regulation data can be accessed, please send an email to cohort.tempo@inserm.fr. Anonymized data can only be shared after explicit approval of the French national committee for data protection for approval (*Commission Nationale de l’Informatique et des Libertés*, CNIL).
