# Supplementary material for: Factors associated with the use of cannabis for self-medication by adults: data from the French TEMPO cohort study
Source: J Cannabis Res. 2024 Apr 10;6:19. doi: 10.1186/s42238-024-00230-2 (PMC11005193; doi:10.1186/s42238-024-00230-2)
Supplement: Supplementary file 1 — Supplementary Material 1. [file 42238_2024_230_MOESM1_ESM.docx]

**Additional file 1. Reasons and forms selected from the questionnaire to define medical use of cannabis**

|  | n (%) | Initial self-medication definition n = 36 | Sensitivity analysis  n = 136 | |
| --- | --- | --- | --- | --- |
| *Reasons to use cannabis* | | | |  |
| **To manage stress** | 21 (6.1%) |  |  | |
| **To manage anxiety** | 13 (3.8%) |  |  | |
| **To manage headaches, migraines** | 4 (1.2%) |  |  | |
| **To treat chronic pain** | 4 (1.2%) |  |  | |
| **For depression** | 3 (0.9%) |  |  | |
| **For muscle spasms** | 3 (0.9%) |  |  | |
| **To manage nausea** | 3 (0.9%) |  |  | |
| **For loss of appetite** | 2 (0.6%) |  |  | |
| **For muscle stiffness** | 2 (0.6%) |  |  | |
| **To treat epileptic seizures** | 0 (0%) |  |  | |
| **For tremors** | 1 (0.3%) |  |  | |
| **To prevent vomiting** | 1 (0.3%) |  |  | |
| **To feel well** | 113 (32.8%) |  |  | |
| **To forget about life’s problems** | 28 (8.1%) |  |  | |
| **To fill a void** | 12 (3.5%) |  |  | |
| **To be happy** | 10 (2.9%) |  |  | |
| **To do as the others do** | 52 (15.1%) |  |  | |
| **To fit in with a group** | 33 (9.6%) |  |  | |
| **To go towards the others** | 3 (0.9%) |  |  | |
| **To spend a good evening with friends** | 131 (38.0%) |  |  | |
| **To have fun** | 122 (35.4%) |  |  | |
| **To get high** | 54 (15.7%) |  |  | |
| **To stimulate oneself, to find energy** | 3 (0.9%) |  |  | |
| **To increase cerebral capacities** | 3 (0.9%) |  |  | |
| **To improve work capacity** | 2 (0.6%) |  |  | |
| *Forms of cannabis use* | | | |  |
| **Medication (Cesamet, Epidyolex, Marinol, Sativex)** | 3 (0.9%) |  |  | |
| **Oil** | 3 (0.9%) |  |  | |
| **Capsules** | 1 (0.3%) |  |  | |
| **Cream, pomade** | 0 (0.0%) |  |  | |
| **Herb (marijuana, ganja, weed)** | 227 (67.0%) |  |  | |
| **Resin (hashish, hash, pot** | 169 (49.9%) |  |  | |
| **Food (cakes, butter, ...)** | 19 (5.6%) |  |  | |
| Total of selected reasons or forms |  | 16 | 20 | |
