# Supplementary material for: Factors associated with the use of cannabis for self-medication by adults: data from the French TEMPO cohort study
Source: J Cannabis Res. 2024 Apr 10;6:19. doi: 10.1186/s42238-024-00230-2 (PMC11005193; doi:10.1186/s42238-024-00230-2)
Supplement: Supplementary file 2 — Supplementary Material 2. [file 42238_2024_230_MOESM2_ESM.docx]

**Additional file 2. Factors associated with cannabis use for self-medication by logistic regression including negative life events (with and without cannabis use trajectory) (TEMPO cohort study, 2020-2021, France, n = 345)**

|  | Multivariate imputed^1^ model | | | |
| --- | --- | --- | --- | --- |
| Variable | OR [95% CI]^2^ | p-value | OR [95% CI]^2^ | p-value |
| **Socioeconomic position** |  |  |  |  |
| Intermediate or high SEP | reference |  | reference |  |
| Lower SEP | 1.76 [0.77 to 4.03] | 0.178 | 1.79 [0.81, 3.96] | 0.150 |
| **Cannabis use trajectory** |  |  |  |  |
| Experimentation | reference |  |  |  |
| Decreasing consumption | 4.05 [1.63 to 10.1] | **0.003** |  |  |
| High consumption | 6.21 [2.00 to 19.3] | **0.002** |  |  |
| **Musculoskeletal disorders** |  |  |  |  |
| No | reference |  | reference |  |
| Yes | 2.51 [1.08 to 5.81] | **0.032** | 2.03 [0.93, 4.45] | 0.077 |
| **Insomnia** |  |  |  |  |
| No | reference |  | reference |  |
| Yes | 1.98 [0.75 to 5.22] | 0.167 | 1.91 [0.76, 4.81] | 0.168 |
| **Smoking status** |  |  |  |  |
| Non-smoker | reference |  | reference |  |
| Regular, occasional or ex-tobacco smoker | 2.58 [0.89 to 7.53] | 0.082 | 4.02 [1.47, 11.0] | **0.007** |
| **Repeating a grade** |  |  |  |  |
| No | reference |  | reference |  |
| Yes | 1.00 [0.44 to 2.30] | 1.000 | 1.04 [0.47, 2.32] | 0.919 |
| **Violence during childhood** |  |  |  |  |
| No | reference |  | reference |  |
| Yes | 1.15 [0.49 to 2.68] | 0.749 | 1.32 [0.58, 2.99] | 0.509 |
| **Parental divorce before 17 years old** |  |  |  |  |
| No | reference |  | reference |  |
| Yes | 3.07 [0.86 to 11.0] | 0.085 | 4.42 [1.29, 15.1] | **0.018** |
| **Parental depression before 17 years old** |  |  |  |  |
| No | reference |  | reference |  |
| Yes | 2.56 [0.93 to 7.05] | 0.070 | 2.54 [0.95, 6.77] | 0.063 |

^1^ Model imputed with MICE

^2^ OR = Odds Ratio, 95% CI = Confidence Interval
